# Effects of Acute Dengue Infection on Sperm and Virus Clearance in Body Fluids of Men

**DOI:** 10.3201/eid2806.212317

**Published:** 2022-06

**Authors:** Joffrey Mons, Dominique Mahé-Poiron, Jean-Michel Mansuy, Hélène Lheureux, Delphine Nigon, Nathalie Moinard, Safouane Hamdi, Christophe Pasquier, Nathalie Dejucq-Rainsford, Louis Bujan

**Affiliations:** Groupe Hospitalier Sud Réunion, St. Pierre, France (J. Mons, H. Lheureux);; Institut National de la Santé et de la Recherche Médicale, Rennes, France (D. Mahé-Poiron, N. Dejucq-Rainsford);; Centre Hospitalier-universitaire, Toulouse, France (J.-M. Mansuy, N. Moinard, S. Hamdi, C. Pasquier, L. Bujan);; Institut National de la Santé et de la Recherche Médicale, Toulouse-Montpellier, France (N. Moinard, S. Hamdi, L. Bujan)

**Keywords:** dengue, dengue virus, DENV, sexually transmitted infections, febrile illnesses, sperm, semen, vector-borne infections, mosquito-borne diseases, arboviruses, viruses

## Abstract

We investigated the effects of dengue virus (DENV) on semen using samples collected 7, 15, 30, 60, and 90 days after symptom onset from 10 infected volunteers on Réunion Island. We assessed characteristics of semen and reproductive hormones and isolated motile spermatozoa from semen. We assayed semen for DENV using reverse transcription PCR and searched for DENV RNA by virus isolation in Vero E6 cell cultures. Four volunteers had >1 DENV RNA-positive semen samples; 2 volunteers had DENV RNA–positive semen at day 15 and 1 at day 30. No motile sperm were DENV positive. After exposure to positive semen, few Vero E6 cells stained positive for DENV antigens, indicating low levels of replicative virus. We found DENV had shorter duration in semen than in blood. These findings support the possibilities that DENV is sexually transmissible for a short period after acute dengue illness and that acute dengue induces reversible alterations in sperm.

Dengue is a mosquitoborne viral disease common in regions with tropical climates. Worldwide annual dengue incidence is estimated at ≈100 million diagnosed and ≈300 million asymptomatic infections. To date, it is estimated that >55% of the population worldwide is exposed to dengue virus (DENV), an RNA virus from the genus *Flavivirus* of the Flaviviridae family. The disease represents a global health issue because it is endemic in >100 countries and the World Health Organization listed dengue as a top 10 disease threat in 2019 (https://www.who.int/news-room/spotlight/ten-threats-to-global-health-in-2019). Climate change and increased circulation of *Aedes albopictus* mosquitoes in temperate countries mean that dengue incidence is being forecast to increase and extend to regions not previously affected.

Most dengue-infected persons are asymptomatic or develop a mild form of the disease with common signs and symptoms resembling those of influenza, such as fever, retroocular pain, headache, rash, muscle and joint pain, nausea, vomiting, and fatigue. However, a small proportion of infections progress to severe illness that can cause rapid onset of capillary leakage leading to bleeding, thrombocytopenia, and rapid shock. Severe dengue can be fatal, and it is often impossible to predict the progression from mild infection to severe dengue (*1*). 

Dengue virus (DENV), which has 4 antigenically distinct genotypes (1–4), is transmitted to humans by bites from female *Aedes* mosquitoes, usually *Ae. aegypti* and *Ae. albopictus*. In addition, like Zika virus (ZIKV), DENV transmission by routes other than mosquitoes has been documented. Vertical transmission of DENV from pregnant women to fetuses, associated with increased rates of preterm birth, low birthweight, and miscarriage, has been reported (*2*–*5*). DENV has been found in breast milk, and transmission to infants through breast-feeding has been reported (*6*). Transmission has also been reported through blood products by mucocutaneous contact or needlestick injury during patient care or laboratory work (*7*,*8*) and by blood transfusion (*9*,*10*) or organ (*11*) or blood stem cell (*12*) transplants.

Sexual transmission of DENV was recently reported in a male patient returning to Spain from Cuba and Puerto Rico who developed dengue symptoms at the time of arrival, 7–10 days after having unprotected sexual intercourse with a male partner, who also developed dengue (*13*). Reverse transcription PCR (RT-PCR) testing of semen samples found identical genomic DENV viral sequences in both partners. Probable woman-to-man sexual transmission of DENV in South Korea was reported elsewhere (*14*). Two studies on DENV in semen have yielded discrepant results. A case report described detection of DENV RNA in semen 9, 24, and 37 days after symptom onset, but viral isolation using cell culture was unsuccessful (*15*). In contrast, another study documented failure to detect DENV RNA in semen from 5 DENV-infected men studied 3–5 days after fever onset (*16*). We conducted a prospective longitudinal study to investigate the relationships between whole blood, serum, urine, and semen DENV RNA viral loads over time, to identify characteristics of DENV in semen and within the different semen compartments, and to determine semen characteristics and reproductive hormone levels up to 90 days after infection to research the effects of DENV infection on human reproductive function.

## Methods

### Study Design and Subjects

For the study, we included men 18–45 years of age in France’s overseas department of Réunion Island (located in the Indian Ocean) who had diagnosed DENV-2 infection (DENV RNA detected in blood serum). We excluded men who had other acute illnesses, were unable to provide a semen sample >1.5 mL, had an ejaculation disorder, or tested negative for DENV RNA in serum or urine. Patients attended follow-up visits 7, 15, 30, 60, and 90 days after symptom onset (Appendix). We collected whole blood, serum, urine, and semen samples at each visit.

After a press information campaign in Réunion Island, 12 male patients with clinical symptoms of acute dengue virus infection were recruited through physicians and underwent a DENV RNA test in serum. Of these 12 men, we excluded 1 with a negative DENV RNA test and 1 with very low semen volume. We enrolled the remaining 10 men, who were diagnosed with acute symptomatic DENV infection and were positive for DENV RNA, in Saint-Pierre University Hospital, Réunion Island. This area has been an officially designated DENV-2 outbreak area since the beginning of 2018. 

The study was registered at ClinicalTrials.gov (NCT03612609) and approved by the institutional ethics review board (CPP Sud-Méditerranée II). All volunteers gave written informed consent and received compensation (€400) for their participation. At each visit, participants completed a questionnaire about any unusual events since their previous visit to the laboratory.

### Specimen Collection and Analysis

Volunteers provided 46 semen samples by masturbation after a recommended 3–6 d abstinence period. We processed the samples within 1 h after ejaculation for analysis. We centrifuged the samples at 600 × *g* and obtained seminal plasma and whole semen cells from a 200 μL semen aliquot and froze the products at −80°C. We performed semen analysis according to World Health Organization guidelines (Appendix).

At 7 and 15 days after symptoms began, we processed an aliquot of semen to isolate spermatozoa cell populations (80% fraction and swim-up fraction) according to methods used for HIV-infected men, published elsewhere (*17*) (Appendix). We performed DENV RNA searches on 40% and 80% fractions obtained from DENV RNA-positive seminal plasma or semen cells samples. We collected morning urine, whole blood, and serum samples at Saint-Pierre University Hospital, Réunion Island, and froze the samples until DENV RT-PCRs were performed at Toulouse University Hospital (Toulouse, France).

### Hormonal Analyses

We assessed serum AMH (anti-Müllerian hormone), FSH (follicle-stimulating hormone), LH (luteinizing hormone), and testosterone levels using Cobas 8000e602 automated immunoassay (Roche Diagnostics, https://www.roche.com). We quantified serum inhibin B levels in duplicate using a manual ELISA assay (Ansh Labs, https://www.anshlabs.com) with a quantification limit of 4.6 pg/mL.

### Virologic Methods—DENV RNA Detection

We extracted RNA from whole blood, serum, urine, and semen fractions with the MagNA Pure 96 instrument using the DNA and viral NA small volume kit (Roche Diagnostics) with 200 μL input and 100 μL output minimum volumes. For semen cell fractions, we adjusted input volume to 2 × 10^6^ cells. We detected DENV RNA using a homemade 1-step real-time RT-PCR triplex protocol to specifically detect DENV, ZIKV, or chikungunya (CHIKV) RNA (*18*). We added 15 μL of extracted RNA to a 45 μL amplification containing 140 nmol/L of each primer; 100 nmol/L each of GAPDH-LC670, DENV-LC610, and ZIKV-FAM TaqMan probes; 45 nmol/L of CHIKV-Cyan500 TaqMan probe; and 1 μL of enzyme (Superscript III Platinum One-step RT-PCR kit, ThermoFisher Scientific, https://www.thermofisher.com). We used a LightCycler 480 Thermocycler (Roche Diagnostics) for amplification and detection with reverse transcription at 52°C for 20 min, melting at 95°C for 2 min, followed by 45 cycles with denaturation at 95°C for 15 s, hybridization at 55°C for 45 s, elongation at 68°C for 20 s, and finally, cooling at 40°C for 30 s.

### Virological Methods—Isolation of Infectious DENV

We incubated semen samples from the 2 donors with the highest DENV RNA levels in seminal plasma, cell fractions, or both, or from an uninfected donor on subconfluent Vero cells, as described elsewhere (*19*), with some modifications. We incubated 200 μL seminal plasma diluted 5-fold in culture medium or cell pellets diluted in 900 μL medium for 5 h with subconfluent Vero E6 cells cultured onto glass coverslips in 24-well plates (250 μL diluted seminal plasma/well or 300 μL diluted cells/well). We washed out seminal plasma and cell pellets and cultured Vero cells with fresh medium for 6 d. We submitted Vero cells from this first passage to immunofluorescence using the flavivirus group antigen antibody, clone D1-4G2-4-15 (Millipore Sigma, https://www.emdmillipore.com), to search for DENV-positive cells, as described elsewhere (*20*). We observed no labeling in Vero cells incubated with uninfected or infected semen samples. We performed a second passage by adding 800 μL of supernatants collected from the first passage (i.e., Vero cells cultured for 6 d after direct contact with semen fractions) to fresh Vero cells, which we also cultured for 7 d. At the end of this second round of amplification, we submitted Vero cells to immunofluorescence using the 4G2 antibody. The negative controls (i.e., mock-exposed Vero cells and Vero cells exposed to semen from uninfected donors) showed no labeling, ensuring the specificity of DENV detection. We transferred all frozen samples to the Germethèque biobank (BB-0033-00081; https://www.chu-toulouse.fr/-germetheque-centre-de-ressources-biologiques) and centralized data from case report forms at Toulouse University Hospital.

### Statistical Analysis

Data are presented as medians and interquartile ranges, quartiles 1–3, and as boxplots for graphic representation. We recorded differences between day 7 after symptom onset and days 15, 30, 60, and 90 for sperm characteristics using the Wilcoxon signed rank-sum test and for hormone values. Because there were multiple comparisons, we used a Bonferroni correction with p<0.0125 considered significant. We considered p<0.05 significant if there was no Bonferroni correction. We used Stata IC15 software (StataCorp, LLC, https://www.stata.com) to perform statistical analyses.

## Results

We included 10 DENV-2–positive men in the study. Mean age was 33.4 [SD 6.1] years; 7 of the men were Creole and 3 were White. All were RNA-negative for ZIKV and CHIKV in blood. All 10 patients reported febrile episodes, muscle aches, joint pain, and asthenia; 9 reported headaches, and 5 had skin rash or conjunctivitis. However, all disease signs and symptoms were mild, and no patient was hospitalized. We found no clinical signs of orchitis. We did not explore sexual function in this study, and no patient spontaneously reported sexual dysfunction.

Four men had no children, and 6 had 1–3 children. Only 1 man had previously provided a sperm sample for fertility investigation. No patient had a history of genital infection or previous genital surgery. One patient had diabetes and 1 had Crohn’s disease. All patients experienced a febrile episode with a median duration of 4 days (range 3–5 days). Two patients were moderate smokers (3 and 10 cigarettes/d). All patients worked but we identified no occupational risk to fertility. We prospectively followed 9 patients for 90 days; 1 patient withdrew after his first visit on Day 7 (no reason given). We included in our statistical analysis only data from the 9 patients who attended all visits.

### DENV RNA Detection

**Figure 1:**
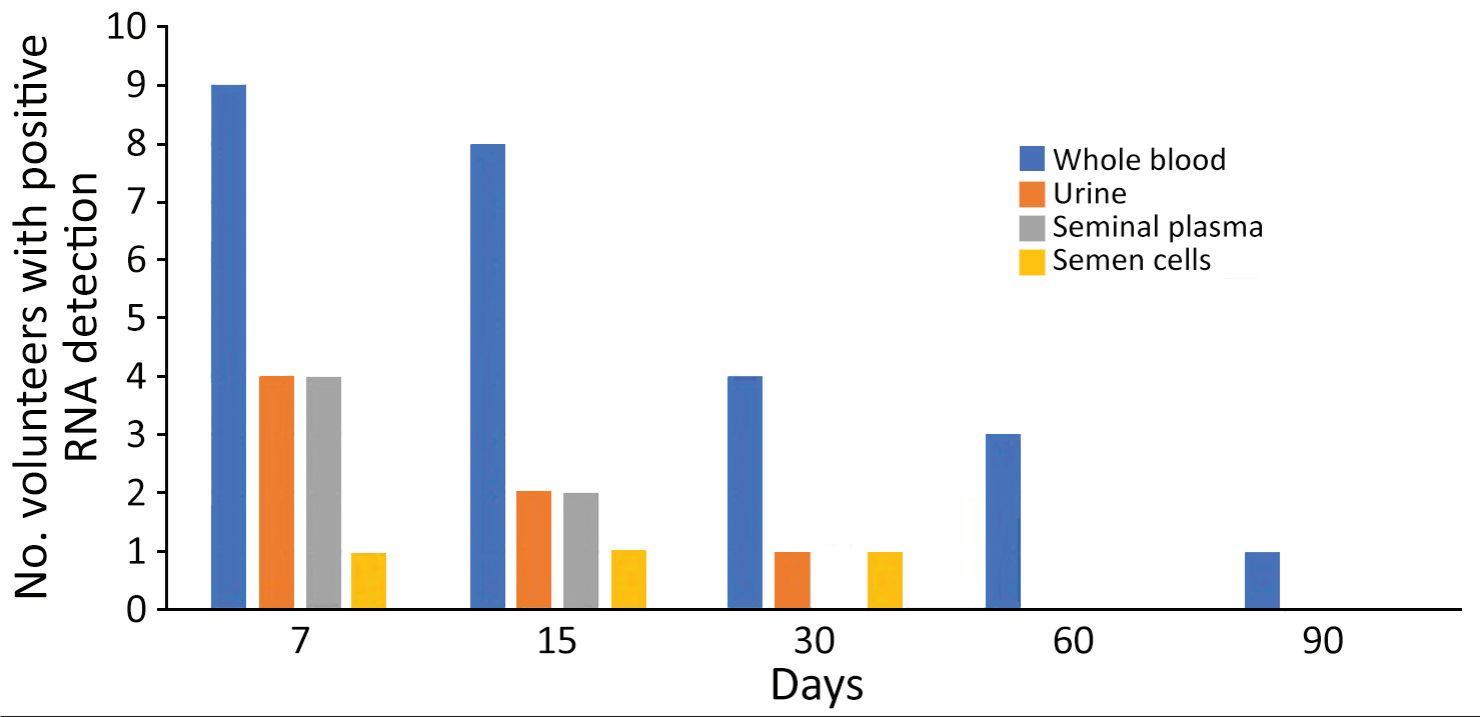
Frequency of dengue virus RNA detection in different fluids in 9 volunteers from Réunion Island with dengue virus infection in a study of virus clearance and effects on reproductive function, according to time points (days) after onset of signs and symptoms.

During follow-up, we found that all but 1 volunteer had a DENV RNA-positive result in >1 body fluid sample. Among whole blood samples, 25/46 (54.3%) were DENV RNA-positive (Ct range 27.95–42.89), but the number of volunteers with positive whole blood samples decreased over time. Only 1 volunteer had a positive whole blood sample at 90 days (Figure 1; Appendix Table). Four volunteers had DENV RNA-positive urine specimens from >1 visit and a total of 7/46 (15.2%) urine specimens were DENV RNA-positive (Ct range 32.66–38.33). One volunteer had a positive urine specimen at day 30 but no volunteer tested positive after day 30.

Among the 10 volunteers supplying seminal plasma or semen samples at day 7, a total of 4 (40%) had >1 DENV RNA-positive result. We never detected DENV RNA in semen or its fractions in 6 (60%) volunteers, including 1 tested only on Day 7. We found DENV RNA in both seminal plasma and semen cell samples from 2 volunteers and only in seminal plasma samples from 2 others. We systematically confirmed all RNA detections by retesting the samples (Ct range 33.9–41.6); 3/30 (10%) semen cell samples and 6/30 (20%) seminal plasma samples were DENV RNA-positive. Two of 9 volunteers had positive seminal plasma and semen cells samples at day 15 and 1/9 had a positive semen cell result at day 30. We isolated spermatozoa fractions from all but 2 semen samples at day 7 and from all semen samples at day 15. We found no 80% fractions containing only spermatozoa positive for DENV RNA and two 40% fractions among 12 samples tested that were DENV RNA-positive.

### DENV Isolation

**Figure 2:**
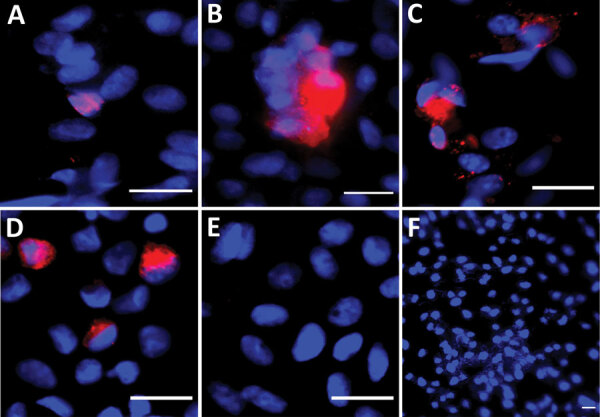
Immunofluorescent images from Vero E6 cells exposed to semen samples obtained at day 7 after symptom onset from volunteers from Réunion Island with dengue virus (DENV) infection in a study of virus clearance and effects on reproductive function. A) Seminal plasma (patient 6); B) whole semen cells (patient 3); C) cells from 40% fraction obtained after semen preparation (patient 6); D) DENV-1 infected Vero E6 cells (positive control) (multiplicity of infection 0.01 for 3 days); E) noninfected Vero E6 cells; F) Vero E6 cells inoculated with noninfected semen (negative controls). VeroE6 cells were inoculated with the semen fractions indicated from DENV-infected (A, B, C) or uninfected patients (E) and cultured for 7 days (first passage). Images were made after detection of DENV envelop protein (DENV-E) from a second passage on VeroE6 of culture supernatants collected after the first passage. Red indicates DENV-E, blue indicates DAPI staining. Scale bars indicate 20 μm.

We attempted to isolate DENV from semen samples displaying the highest viral loads: whole semen cells from patient 3 and seminal plasma and 40% fractions obtained from patient 6 after isolation from semen. We detected a few DENV-positive Vero E6 cells after exposure to semen samples using immunocytofluorescence against the viral envelope (Figure 2). However, we detected no DENV RNA in the supernatants of the semen-exposed Vero E6 cultures (data not shown).

**Figure 3:**
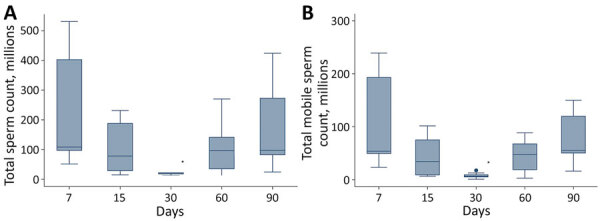
Semen characteristics of 9 dengue virus–infected volunteers from Réunion Island at specific time points (days) after onset of signs and symptoms from dengue virus infection. A) Total sperm count (millions/ejaculate); B) total motile sperm count (millions/ejaculate). Horizontal lines within boxes indicate medians; box tops and bottoms indicate interquartile ratios; error bars indicate minimums and maximums. Significant value (*p<0.0125) between day 7 and subsequent days

Median sperm count, total sperm count, and total motile sperm count significantly decreased by day 30 (p<0.0125); median sperm count was only 15% and total sperm count only 19% of sperm production at day 7. The percentage of normal sperm was lowest at day 30 but this decrease did not reach statistical significance, probably because of the small number of volunteers studied (Table 1; Figure 3). We found no statistical differences between results at the different follow-up times for any of the hormones studied; however, the testosterone/LH ratio was significantly lower at day 7 than at days 15 and 30 (p<0.0125) (Table 2).

**Table 1:** Semen characteristics of 9 dengue virus–infected volunteers according to time points after symptom onset, Réunion Island*

Characteristic	Median value (interquartile range)				
	Day 7	Day 15	Day 30	Day 60	Day 90
Volume, mL	1.8 (1.7–2.2)	3 (2–6)	2.2 (1.6–2.5)	2.5 (2–3.8)	2.4 (1.8–2.9)
Sperm count, millions/mL	60 (46–72)	55 (13–70)	9 (6–20)†	25 (20–49)	54 (22–80)
Total sperm count, millions/ejaculate	108 (96–403.2)	78 (27.3–189)	20.7 (16.8–22.5)†	96 (34–142.1)	97.2 (81.4–273.6)
Total motile sperm count, millions/ejaculate	53.76 (48.96–193.54)	34.32 (8.51–75.6)	6.53 (4.76–9.75)†	47.52 (18.36–68.16)	55.35 (49.92–120.38)
Progressive motility, %	47 (45–55)	43 (40–44)	34 (26–37)	53 (45–59)	53 (50–67)
Vitality, %	86 (75–91)	80 (71–83)	81 (75–88)	87 (84–90)	87 (83–91)
Normal, %	15 (10–17)	14 (11–19)	10 (9–14)	15.5 (11–18)	16.5 (11.5–20)

*Ten volunteers were recruited, but only 9 completed follow-up. †p<0.0125 between day 7 and days 15, 30, 60, 90.

**Table 2:** Hormone values in serum samples of 9 dengue virus–infected volunteers at different time points after symptom onset, Réunion Island*

Hormones†	Median value (interquartile range)				
	Day 7	Day 15	Day 30	Day 60	Day 90
LH, IU/L	5.1 (4.2–6.7)	3.8 (3.3–7.3)	4 (2.9–5.3)	3.8 (3.4–4.8)	5 (3.6–6.6)
FSH, IU/L	3.6 (2.5–5)	3.5 (2.6–6)	3.9 (2–4.7)	3.0 (2.1–4.1)	3.3 (2–4.8)
Inhibin, pg/mL	157 (141–230)	163 (133–249)	179 (147–270)	167 (135–215)	179 (160–204)
AMH, ng/mL	6.4 (5.5–6.6)	5.9 (5.5–6.4)	6.9 (6.1–7.8)	7.1 (6.6–9.4)	7.6 (5.5–8.8)
Testosterone, ng/dL	333 (321–403)	479 (353–645)	507 (312–570)	438 (302–557)	450 (394–511)
Testosterone/LH ratio	72 (20–106)	114 (56–191)‡	113 (61–181)‡	106 (47–199)	89 (49–162)

*Ten volunteers were recruited, but only 9 completed follow-up; AMH, anti-Müllerian hormone; FSH, follicle-stimulating hormone; LH, luteinizing hormone. †Normal range values: LH, 1.7–8.6UI/l; FSH, 1.5–12.4 IU/l; inhibin, 152–174 pg/mL; testosterone, 260–1,000 ng/dL. ‡p<0.0125 between day 7 and days 15, 30, 60, and 90.

## Discussion

Our prospective study provides results from a longitudinal assessment of different biologic samples (whole blood, urine, and semen) and reports semen and reproductive hormones characteristics after acute DENV infection in men. We also document the detection and clearance of DENV RNA in different semen fractions and the presence of replicative virus, including in motile spermatozoa fractions, generally used in assisted reproductive procedures.

Ten symptomatic patients provided first samples 7 days after symptom onset. We recruited these patients after they were diagnosed with DENV infection through positive results from molecular testing during the 2018–2019 dengue epidemic on Réunion Island. Whole blood samples were DENV RNA-positive for all but 1 patient, who was negative at days 7 and 15 after symptom onset. Viremia remained in 2 patients at day 60 and in 1 at day 90 after symptom onset. The duration of DENV viremia has been estimated at ≈9.1 days (95% CI 4.4–13.9 days) (*21*). DENV RNA detection was more sensitive and the diagnostic window was probably longer for whole blood than serum samples (*22*). Prolonged viremia of 18–80 days has been reported in hematopoietic stem cell transplant recipients with hematological malignancies (*23*). However, the patient with the longest duration of viremia in our study had no disease. We detected DENV RNA less frequently in urine than in whole blood; only 40% of patients had a positive urine result. One urine sample was DENV RNA-positive at day 30 in a patient whose whole blood sample was positive at the same time, but no later urine samples were positive.

Two previous studies looked for DENV in semen from infected men, with discrepant results. In a case report of a man returning from Thailand, DENV-2 RNA was detected in semen (Ct 24–31.8) 9, 24, and 37 days after dengue symptom onset, but no infectious virus could be detected (*15*). In contrast, in another study from Singapore of 5 DENV-infected men, DENV RNA was not detected in semen 3–5 days after fever (*16*). In our study, 4 (40%) of the 10 infected men tested had >1 DENV RNA-positive semen samples. Seminal plasma was more often positive than semen cell pellets. Positivity in semen was independent of results from urine, suggesting that infection of these fluids originated in different tissues. However, unlike in some other viruses, such as ZIKV (*19*), DENV positivity in semen was always associated with whole-blood positivity in our study. Of note, semen was DENV positive 30 days after symptom onset in our study, a duration similar to that reported elsewhere (*15*).

Other flaviviruses, such as hepatitis C virus or ZIKV, have been found in semen (*19*,*24*). Although DENV is a more widespread flavivirus than ZIKV, data on the tropism and effects of DENV in the male genital tract are scarce. Four cases of acute scrotal edema associated with DENV infection have been reported, but their mechanisms were not elucidated (*25*,*26*). Although ZIKV actively replicates in the human testis ex vivo and persists in this organ (*20*,*27*), it is unknown whether DENV can infect the human testis, an established viral reservoir (*28*). In vitro DENV was able to infect a human Sertoli cell line, but less efficiently than ZIKV (*29*). In nonhuman primates infected with DENV, viral proteins were observed in the seminal vesicles and prostate but not in the testis (*30*). The origin of DENV in semen requires further studies. Because detection of DENV RNA in semen does not imply that semen is infectious, we attempted to isolate replication-competent virus from semen using Vero E6 cells. Only a few Vero E6 cells positive for DENV envelope were observed after contact with seminal plasma or cell pellets (40% fraction), suggesting a low level of replicative virions in those samples. This finding is in line with a relatively low level of DENV RNA in semen, as suggested by the Ct values in the range of 33.9 to 41.6. DENV RNA detection was negative in the Vero E6 culture supernatants, which might be because of a replication level below the detection limit. Further studies will be needed to confirm the isolation of replication-competent virus in semen from DENV-infected men.

Although dengue is a common infection worldwide, only 2 cases of sexually transmitted DENV have been reported (*13*,*15*), unlike ZIKV, for which sexual transmission has been reported in 14 countries outside the epidemic area. Those results may reflect a lower viral load in semen for DENV. In addition, some evidence supports a shorter duration of DENV excretion in semen compared with ZIKV; ZIKV RNA has been detected in semen up to 414 days after symptom onset (*31*), and replicative virus has been shown to persist in seminal cells up to 90 days (*27*), whereas for this study we detected DENV RNA in semen no longer than 30 days after symptom onset. Moreover, infectious virus was readily detected in the semen from ZIKV-infected persons, unlike for DENV patients, in whom we observed only sparse counts of infected reporter Vero E6 cells, suggesting a low level of infectious virions in the semen samples.

To better identify the location of DENV within semen, we submitted the collected semen to a density gradient process, which separates the different fractions of semen and isolates sperm cell fractions. Semen pellets (sperm and nonsperm cells) and seminal plasma from our patients were positive for DENV RNA, but not motile spermatozoa cell fractions, indicating that either DENV was not associated with motile spermatozoa or the viral load was below the detection threshold. The sperm processing methods used for assisted reproductive technology therefore seem to be effective for isolating spermatozoa free of DENV virus as they do for several other viruses. Nevertheless, this finding remains to be demonstrated in cases of high DENV shedding in semen, because this technique was not sufficient to remove infectious spermatozoa from the semen of ZIKV-infected men with a high semen viral load (*19*).

In this prospective study, we found that sperm production decreased 30 days after symptom onset. Although we found no significant changes in reproductive hormones, the significant decrease in the testosterone/LH ratio might reflect subtle Leydig cell dysfunction at day 7 after symptom onset. These transient sperm alterations could have resulted from the viral infection itself, from fever, or from both, because all patients self-reported febrile episodes. Although the precise origins of such alterations after DENV infection still need to be identified, it is noteworthy that increased sperm DNA fragmentation has been observed after testicular or epididymal hyperthermia (*32*).

One limitation of our prospective study was the relatively low number of patients and the absence of a control group of noninfected men for the study of semen and hormonal characteristics. Also, this study included men with slight or mild symptoms of dengue infection; no data were available on the effects in men with asymptomatic or severe dengue.

In conclusion, this study provides a longitudinal assessment of the detection and clearance of DENV in different body compartments. We demonstrate that DENV RNA is detected in whole blood longer than in serum and urine and show that DENV RNA was found in semen up to 30 days after symptom onset but was not associated with motile sperm cells. Development of knowledge about the interactions between DENV and the reproductive tract is currently in its infancy. These findings emphasize the need for further studies in this field and also have implications for public health policy, such as contributing to increased diagnostic efficiency and limiting sexual transmission of DENV. Finally, our findings provide information relevant to counseling DENV-infected patients and couples who wish to conceive a child.

Appendix Additional information on effects of acute dengue infection on virus clearance and effects on reproductive function in men

## References

[1] Nedjadi T, El-Kafrawy S, Sohrab SS, Desprès P, Damanhouri G, Azhar E. Tackling dengue fever: Current status and challenges. Virol J. 2015;12:212. 10.1186/s12985-015-0444-8 26645066 PMC4673751

[2] Chye JK, Lim CT, Ng KB, Lim JM, George R, Lam SK. Vertical transmission of dengue. Clin Infect Dis. 1997;25:1374–7. 10.1086/516126 9431381

[3] Thaithumyanon P, Thisyakorn U, Deerojnawong J, Innis BL. Dengue infection complicated by severe hemorrhage and vertical transmission in a parturient woman. Clin Infect Dis. 1994;18:248–9. 10.1093/clinids/18.2.248 8161636

[4] Paixão ES, Teixeira MG, Costa MDCN, Rodrigues LC. Dengue during pregnancy and adverse fetal outcomes: a systematic review and meta-analysis. Lancet Infect Dis. 2016;16:857–65. 10.1016/S1473-3099(16)00088-8 26949028

[5] Pouliot SH, Xiong X, Harville E, Paz-Soldan V, Tomashek KM, Breart G, et al. Maternal dengue and pregnancy outcomes: a systematic review. Obstet Gynecol Surv. 2010;65:107–18. 10.1097/OGX.0b013e3181cb8fbc 20100360

[6] Barthel A, Gourinat AC, Cazorla C, Joubert C, Dupont-Rouzeyrol M, Descloux E. Breast milk as a possible route of vertical transmission of dengue virus? Clin Infect Dis. 2013;57:415–7. 10.1093/cid/cit227 23575200

[7] Chen LH, Wilson ME. Nosocomial dengue by mucocutaneous transmission. Emerg Infect Dis. 2005;11:775. 10.3201/eid1105.040934 15898174 PMC3320385

[8] de Wazières B, Gil H, Vuitton DA, Dupond JL. Nosocomial transmission of dengue from a needlestick injury. Lancet. 1998;351:498. 10.1016/S0140-6736(05)78686-4 9482448

[9] Sabino EC, Loureiro P, Lopes ME, Capuani L, McClure C, Chowdhury D, et al.; International Component of the NHLBI Recipient Epidemiology and Donor Evaluation Study-III. Transfusion-transmitted dengue and associated clinical symptoms during the 2012 epidemic in Brazil. J Infect Dis. 2016;213:694–702. 10.1093/infdis/jiv326 26908780 PMC4747611

[10] Stramer SL, Linnen JM, Carrick JM, Foster GA, Krysztof DE, Zou S, et al. Dengue viremia in blood donors identified by RNA and detection of dengue transfusion transmission during the 2007 dengue outbreak in Puerto Rico. Transfusion. 2012;52:1657–66. 10.1111/j.1537-2995.2012.03566.x 22339201

[11] Rosso F, Pineda JC, Sanz AM, Cedano JA, Caicedo LA. Transmission of dengue virus from deceased donors to solid organ transplant recipients: case report and literature review. Braz J Infect Dis. 2018;22:63–9. 10.1016/j.bjid.2018.01.001 29353669 PMC9425690

[12] Punzel M, Korukluoğlu G, Caglayik DY, Menemenlioglu D, Bozdag SC, Tekgündüz E, et al. Dengue virus transmission by blood stem cell donor after travel to Sri Lanka; Germany, 2013. Emerg Infect Dis. 2014;20:1366–9. 10.3201/eid2008.140508 25062084 PMC4111198

[13] ECDC. Rapid risk assessment—sexual transmission of dengue in Spain. 2019 [cited on 2019 Nov 18]. https://www.ecdc.europa.eu/en/publications-data/rapid-risk-assessment-sexual-transmission-dengue-spain

[14] Lee C, Lee H. Probable female to male sexual transmission of dengue virus infection. Infect Dis (Lond). 2019;51:150–2. 10.1080/23744235.2018.1521004 30318968

[15] Lalle E, Colavita F, Iannetta M, Gebremeskel Teklè S, Carletti F, Scorzolini L, et al. Prolonged detection of dengue virus RNA in the semen of a man returning from Thailand to Italy, January 2018. Euro Surveill. 2018;23:18–00197. 10.2807/1560-7917.ES.2018.23.18.18-00197 29741153 PMC6053624

[16] Molton JS, Low I, Choy MMJ, Aw PPK, Hibberd ML, Tambyah PA, et al. Dengue virus not detected in human semen. J Travel Med. 2018;25:25. 10.1093/jtm/tay023 29672710

[17] Bujan L, Daudin M, Matsuda T, Righi L, Thauvin L, Berges L, et al. Factors of intermittent HIV-1 excretion in semen and efficiency of sperm processing in obtaining spermatozoa without HIV-1 genomes. AIDS. 2004;18:757–66. 10.1097/00002030-200403260-00006 15075510

[18] Mansuy JM, Lhomme S, Cazabat M, Pasquier C, Martin-Blondel G, Izopet J. Detection of Zika, dengue and chikungunya viruses using single-reaction multiplex real-time RT-PCR. Diagn Microbiol Infect Dis. 2018;92:284–7. 10.1016/j.diagmicrobio.2018.06.019 30029808

[19] Joguet G, Mansuy JM, Matusali G, Hamdi S, Walschaerts M, Pavili L, et al. Effect of acute Zika virus infection on sperm and virus clearance in body fluids: a prospective observational study. Lancet Infect Dis. 2017;17:1200–8. 10.1016/S1473-3099(17)30444-9 28838639

[20] Matusali G, Houzet L, Satie AP, Mahé D, Aubry F, Couderc T, et al. Zika virus infects human testicular tissue and germ cells. J Clin Invest. 2018;128:4697–710. 10.1172/JCI121735 30063220 PMC6159993

[21] Busch MP, Sabino EC, Brambilla D, Lopes ME, Capuani L, Chowdhury D, et al.; International Component of the NHLBI Recipient Epidemiology and Donor Evaluation Study-III (REDS-III). Duration of dengue viremia in blood donors and relationships between donor viremia, infection incidence and clinical case reports during a large epidemic. J Infect Dis. 2016;214:49–54. 10.1093/infdis/jiw122 27302934 PMC4907419

[22] Waggoner JJ, Stittleburg V, Natrajan MS, Paniagua-Avila A, Bauer D, Olson D, et al. Sensitive and prolonged detection of dengue virus RNA in whole blood. Am J Trop Med Hyg. 2021;104:1734–6. 10.4269/ajtmh.20-1497 33755591 PMC8103463

[23] de Souza Pereira BB, Darrigo Junior LG, de Mello Costa TC, Felix AC, Simoes BP, Stracieri AB, et al. Prolonged viremia in dengue virus infection in hematopoietic stem cell transplant recipients and patients with hematological malignancies. Transpl Infect Dis. 2017;19:19. 10.1111/tid.12721 28475281

[24] Pasquier C, Daudin M, Righi L, Berges L, Thauvin L, Berrebi A, et al. Sperm washing and virus nucleic acid detection to reduce HIV and hepatitis C virus transmission in serodiscordant couples wishing to have children. AIDS. 2000;14:2093–9. 10.1097/00002030-200009290-00004 11061649

[25] Shamim M, Naqvi SZG. Dengue fever associated with acute scrotal oedema: two case reports. J Pak Med Assoc. 2011;61:601–3. 22204221

[26] Sharda M, Soni A, Nigam H, Singh A, Sharma N. Acute scrotal edema: an atypical manifestation of dengue. J Assoc Physicians India. 2016;64:103–4. 27766820

[27] Mahé D, Bourgeau S, Frouard J, Joguet G, Pasquier C, Bujan L, et al. Long-term Zika virus infection of non-sperm cells in semen. Lancet Infect Dis. 2020;20:1371. 10.1016/S1473-3099(20)30834-3 33248032

[28] Le Tortorec A, Matusali G, Mahé D, Aubry F, Mazaud-Guittot S, Houzet L, et al. From ancient to emerging infections: the odyssey of viruses in the male genital tract. Physiol Rev. 2020;100:1349–414. 10.1152/physrev.00021.2019 32031468

[29] Siemann DN, Strange DP, Maharaj PN, Shi PY, Verma S. Zika virus infects human Sertoli cells and modulates the integrity of the *In vitro* blood-testis barrier model. J Virol. 2017;91:e00623–17. 10.1128/JVI.00623-17 28878076 PMC5660489

[30] Prabandari SA, Arifin E, Saepuloh U, Iskandriati D, Pamungkas J. Dengue virus type 3 (DENV-3) distribution in tissues of pig-tailed macaque (*Macaca nemestrina*) post infection using immunohistochemistry technique. Int J Sci Basic Appl Res. 2017;33:1–9.

[31] Bujan L, Mansuy JM, Hamdi S, Pasquier C, Joguet G. 1 year after acute Zika virus infection in men. Lancet Infect Dis. 2020;20:25–6. 10.1016/S1473-3099(19)30678-4 31876496

[32] Sergerie M, Mieusset R, Croute F, Daudin M, Bujan L. High risk of temporary alteration of semen parameters after recent acute febrile illness. Fertil Steril. 2007;88:970e1–7. 10.1016/j.fertnstert.2006.12.045 17434502

