# Supplementary material for: Effects of Acute Dengue Infection on Sperm and Virus Clearance in Body Fluids of Men
Source: Emerg Infect Dis. 2022 Jun;28(6):1146–53. doi: 10.3201/eid2806.212317 (PMC9155864; doi:10.3201/eid2806.212317)
Supplement: Appendix — Additional information on effects of acute dengue infection on virus clearance and effects on reproductive function in men [file 21-2317-Techapp-s1.pdf]

Article DOI: https://doi.org/10.3201/eid2806.212317

Effects of Acute Dengue Infection on Sperm and Virus Clearance in Body Fluids of Men

Appendix

Methods

Semen analyses

Semen analysis was performed according to WHO guidelines (*1*) on an aliquot (200 µL) of semen. The parameters considered were sexual abstinence (days), ejaculate volume (mL), pH, sperm concentration (SC, 10^6 spermatozoa/mL), spermatozoa vitality (V, %) forward motility (M, a+b: %), total sperm count (volume × SC = TSC, 10^6 spermatozoa/ejaculate) and total motile sperm count (TMSC = TSC × M, 10^6/ejaculate). Sperm cell morphology was assessed according to Auger's modification of the classification of David and colleagues (*1*). All semen analyses were performed by three trained technicians and underwent external quality control.

Sperm processing to isolate spermatozoa fractions

At days 7 and 15, in order to isolate spermatozoa enriched fractions, whole semen was submitted to differential density gradient centrifugation over 40%, and 80% of PureSperm (JCD S.A., Lyon, France) according to a previously published method (*2*). The sperm pellet (80% PureSperm layer) was washed twice with FertiCult IVF medium (JCD S.A., Lyon, France). The 40% fraction contains mostly low motile, dead, or abnormal spermatozoa and seminal non-spermatozoa cells (NSCs).

As the sperm pellet from the 80% fraction could contain rare non-sperm cells, motile spermatozoa from this fraction were further separated by the swim-up method according to a previously published method (*2*). Briefly, the sperm pellet was overlaid with 1.1 mL medium and incubated at 37°C, 5% CO^2 for 60 min in a tube at an angle of 45 degrees to allow the motile spermatozoa to swim up. The swim-up fraction contains only viable motile spermatozoa.

The seminal plasma, the whole semen cells and the different fractions obtained after density gradient preparation and after the swim-up method were collected and stored at –80°C until DENV isolation and genome analyses.

References

<bok>1. World Health Organization. WHO laboratory manual for the examination and processing of human semen, 5th edition. Geneva: World Health Organization; 2010.</bok>

<jrn>2. Bujan L, Daudin M, Matsuda T, Righi L, Thauvin L, Berges L, et al. Factors of intermittent HIV-1 excretion in semen and efficiency of sperm processing in obtaining spermatozoa without HIV-1 genomes. AIDS. 2004;18:757–66. PubMed https://doi.org/10.1097/00002030-200403260-00006</jrn>

Appendix Table. Ct values from reverse-transcription PCR assays to detect dengue.*

		DENV RNA detection, Ct			
Patient	Sampling time post clinical onset	Whole Blood	Urine	Seminal cell pellet	Seminal plasma
1	D7	38.8	32.7	–	-
	D15	41.9	–	–	-
	D30	–	–	–	-
	D60	–	–	–	-
	D90	–	–	–	-
2	D7	36.8	38.3	–	-
	D15	38.8	37.8	–	-
	D30	–	–	–	-
	D60	–	–	–	-
	D90	–	–	–	-
3	D7	27.9	33.8	35.9	36.7
	D15	34.4	36.8	–	40.7
	D30	36.1	38.0	35.0	39.1
	D60	36.7	–	–	-
	D90	–	–	–	-
4	D7	37.6	–	–	38.3
	D15	40.0	–	39.0	38.7
	D30	41.0	–	–	–
	D60	–	–	–	–
	D90	–	–	–	–
5	D7	42.9	–	–	–
	D15	34.9	–	–	–
	D30	36.6	–	–	–
	D60	37.1	–	–	–
	D90	41,2	–	–	–
6	D7	34.4	38.6	–	41.5
7	D7	–	–	–	–
	D15	–	–	–	–
	D30	–	–	–	–
	D60	–	–	–	–
	D90	–	–	–	–
8	D7	35.5	–	–	–
	D15	39.4	–	–	–
	D30	–	–	–	–
	D60	–	–	–	–
	D90	–	–	–	–

Patient	Sampling time post clinical onset	DENV RNA detection, Ct			
		Whole Blood	Urine	Seminal cell pellet	Seminal plasma
9	D7	37.7	–	–	–
	D15	41.1	–	–	–
	D30	–	–	–	–
	D60	–	–	–	–
	D90	–	–	–	–
10	D7	35.7	–	–	42.9
	D15	36.2	–	–	–
	D30	38.1	–	–	–
	D60	39.6	–	–	–
	D90	–	–	–	–

*D, days after symptom onset.

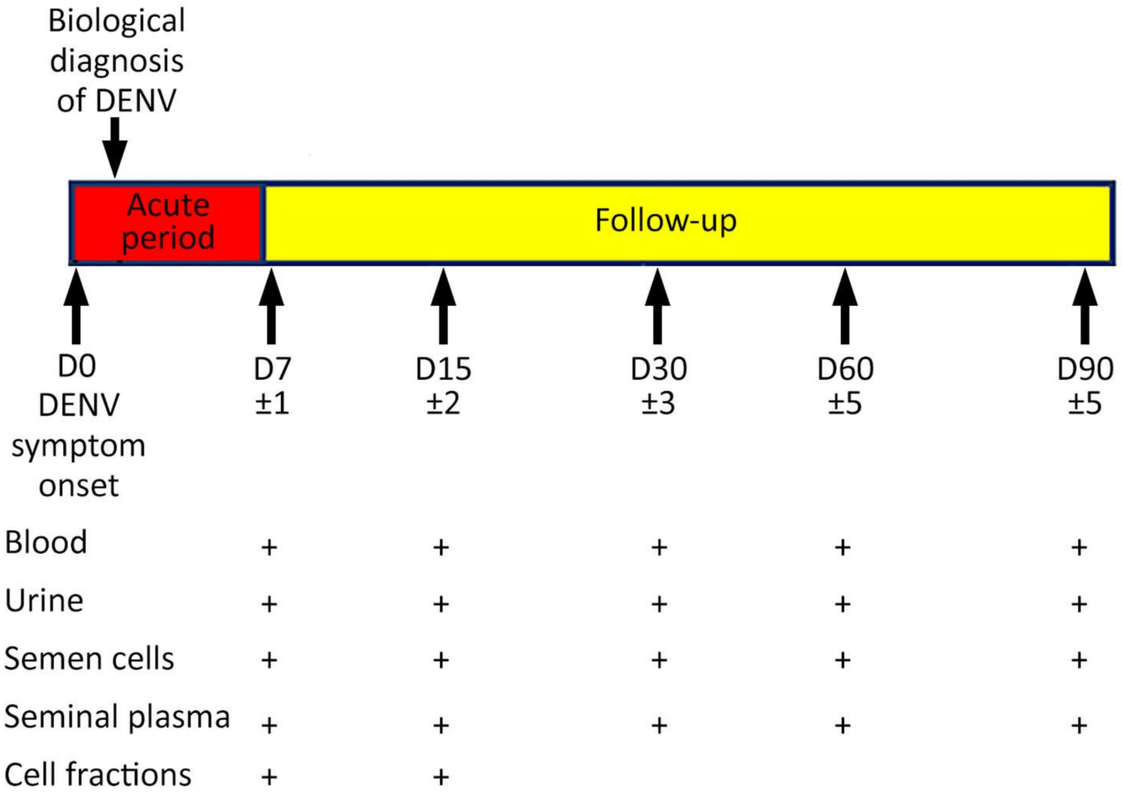

Appendix Figure. Schematic representation of the different sampling times during the follow-up of patients. DO: day of symptom onset following by DENV diagnosis that allows inclusion in the study.
